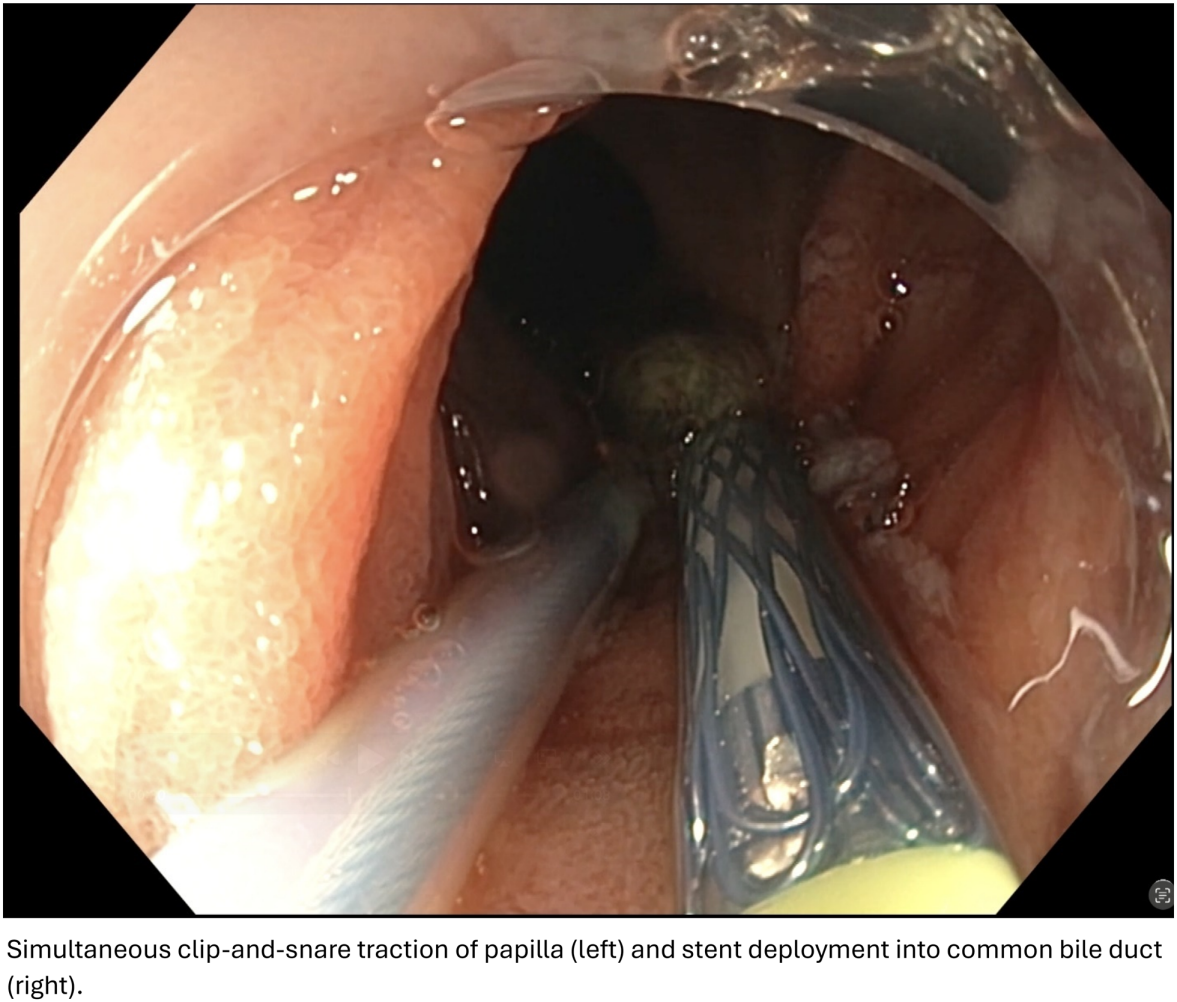# Poster Session I - A77 THE DUAL-CHANNEL CLIP-AND-SNARE TRACTION TECHNIQUE: A NOVEL APPROACH TO BILIARY CANNULATION DURING ENDOSCOPIC RETROGRADE CHOLANGIOPANCREATOGRAPHY IN A PATIENT WITH ROUX-EN-Y ANATOMY

**DOI:** 10.1093/jcag/gwaf042.077

**Published:** 2026-02-13

**Authors:** N Nathoo-Khedri, M Tomaszewski

**Affiliations:** Universite de Montreal, Montreal, QC, Canada; Universite de Montreal, Montreal, QC, Canada

## Abstract

**Background:**

Suboptimal exposure of the ampullary orifice during ERCP renders biliary cannulation technically difficult. Moreover, ERCP in patients with surgically altered gastrointestinal anatomy is challenging. In such cases, traditional guidewire-assisted techniques can be unsuccessful, and innovative approaches may be necessary to facilitate biliary cannulation. Various strategies have been reported in the literature, including endoscopic clipping, submucosal injection, and two-device-in-one-channel methods. The clip-and-snare traction technique has proven to be effective in accomplishing successful biliary cannulation during ERCP in patients with an intradiverticular papilla.

**Aims:**

In this case report, we describe a novel approach to biliary cannulation during ERCP in a patient with Roux-en-Y anatomy: the dual-channel clip-and-snare traction (CST) technique. This novel approach uses a dual channel endoscope allowing simultaneous mobilization of the papilla with a clip and snare to assist in biliary cannulation in patients with altered anatomy.

**Methods:**

Here, we report a successful case of dual-channel biliary cannulation using the CST technique during ERCP in a patient with malignant biliary obstruction and Roux-en-Y anatomy, performed in our centre at Hôpital du Sacré-Coeur in Montreal, Quebec.

**Results:**

An 88-year-old man with Roux-en-Y anatomy presented with jaundice due to malignant compression of the distal common bile duct. ERCP was performed for biliary drainage. A dual channel gastroscope with a distal attachment cap was used to advance to the duodenum via cannulation of the biliopancreatic limb. Upon arrival at the duodenum, a large protruding papilla was observed. Traditional guidewire-assisted cannulation was difficult due to the tangential position of the papillary orifice. Therefore, a MANTIS clip was inserted inferior to the papilla and grasped using a snare inserted through the second channel of the gastroscope. Clip-and-snare traction was performed through the second channel, which enabled successful cannulation of the common bile duct followed by placement of an uncovered metal stent to allow for biliary drainage.

**Conclusions:**

Biliary cannulation can be technically challenging during ERCP in patients with surgically altered gastrointestinal anatomy. The novel clip-and-snare traction technique has proven to be effective in such cases. While the parallel use of CST has been reported to assist in biliary cannulation using a duodenoscope, this case is, to our knowledge, the first report of CST-assisted biliary cannulation using a dual channel gastroscope. The CST method using a dual channel system allows for dynamic mobilization of the papilla due to separate access for devices, thus favoring more efficient biliary cannulation in patients with complex anatomy.

**Funding Agencies:**

None